# Review of Lambda Interferons in Hepatitis B Virus Infection: Outcomes and Therapeutic Strategies

**DOI:** 10.3390/v13061090

**Published:** 2021-06-09

**Authors:** Laura A. Novotny, John Grayson Evans, Lishan Su, Haitao Guo, Eric G. Meissner

**Affiliations:** 1 Division of Infectious Diseases, Medical University of South Carolina, Charleston, SC 29525, USA; novotnyl@musc.edu (L.A.N.); evansjoh@musc.edu (J.G.E.); 2 Division of Virology, Pathogenesis, and Cancer, Institute of Human Virology, Departments of Pharmacology, Microbiology, and Immunology, University of Maryland School of Medicine, Baltimore, MD 21201, USA; LSu@ihv.umaryland.edu; 3 Department of Microbiology and Molecular Genetics, Cancer Virology Program, UPMC Hillman Cancer Center, University of Pittsburgh School of Medicine, Pittsburgh, PA 15261, USA; guoh4@upmc.edu; 4 Department of Microbiology and Immunology, Medical University of South Carolina, Charleston, SC 29425, USA

**Keywords:** hepatitis B virus, interferon lambda, innate immunity, seroclearance

## Abstract

Hepatitis B virus (HBV) chronically infects over 250 million people worldwide and causes nearly 1 million deaths per year due to cirrhosis and liver cancer. Approved treatments for chronic infection include injectable type-I interferons and nucleos(t)ide reverse transcriptase inhibitors. A small minority of patients achieve seroclearance after treatment with type-I interferons, defined as sustained absence of detectable HBV DNA and surface antigen (HBsAg) antigenemia. However, type-I interferons cause significant side effects, are costly, must be administered for months, and most patients have viral rebound or non-response. Nucleos(t)ide reverse transcriptase inhibitors reduce HBV viral load and improve liver-related outcomes, but do not lower HBsAg levels or impart seroclearance. Thus, new therapeutics are urgently needed. Lambda interferons (IFNLs) have been tested as an alternative strategy to stimulate host antiviral pathways to treat HBV infection. IFNLs comprise an evolutionarily conserved innate immune pathway and have cell-type specific activity on hepatocytes, other epithelial cells found at mucosal surfaces, and some immune cells due to restricted cellular expression of the IFNL receptor. This article will review work that examined expression of IFNLs during acute and chronic HBV infection, the impact of IFNLs on HBV replication in vitro and in vivo, the association of polymorphisms in IFNL genes with clinical outcomes, and the therapeutic evaluation of IFNLs for the treatment of chronic HBV infection.

## 1. Hepatitis B Virus 

Hepatitis B virus (HBV) belongs to the *Hepadnaviridae* family, a group of enveloped, hepatotropic DNA viruses that infect mammals and birds with species-specific tropism [1]. After HBV establishes a chronic infection (as reviewed in [2]), the HBV covalently closed circular DNA genome (cccDNA) resides indefinitely as a mini-chromosome within the nuclei of infected hepatocytes and is a stable template for synthesis of HBV transcripts and nascent virion production [2,3,4,5]. During chronic infection, HBV replication within hepatocytes is largely unrestricted, in part as a consequence of the liver’s well-established pre-disposition to antigenic tolerance [6,7,8]. 

Chronic HBV infection causes significant morbidity and mortality worldwide by inducing cirrhosis and hepatocellular carcinoma that result in nearly 1 million deaths per year [9,10,11]. Currently, there are no approved therapies that reliably eliminate or silence cccDNA once chronic infection is established [12,13,14]. Although nucleos(t)ide reverse transcriptase inhibitors (NRTIs) reduce HBV DNA by inhibiting the HBV polymerase, thereby lowering hepatic inflammation and the risk of progressive liver disease, pre-made cccDNA is largely unaffected by NRTI therapy [15]. As such, viral protein production persists during therapy with NRTIs, including production of the HBV surface antigen (HBsAg), a serologic marker of active HBV infection. In addition, long-term NRTI therapy can potentially engender HBV resistance and cause bone and renal side effects which are difficult to manage clinically, underscoring the need for new HBV therapies.

The fate of HBV after an initial infection is highly contingent upon the maturity of the host immune system. Most adults can clear acute HBV infection by cytopathic and/or non-cytopathic mechanisms. This leads to clearance of serum HBV DNA and HBsAg, appearance of antibodies specific for HBsAg, and cessation of de novo viral production. Patients with spontaneous HBV clearance do not have an increased longitudinal risk of the hepatic sequelae associated with chronic infection [6,9,16]. Although there is little direct evidence demonstrating hepatic cccDNA in patients who have spontaneously recovered, HBV can reactivate when immunosuppressive medications are given, providing indirect evidence that intrahepatic cccDNA can persist after spontaneous clearance [17,18,19]. In contrast, neonates or infants infected with HBV typically develop a chronic lifelong infection and are unable to clear cccDNA or virions [6,9]. Understanding the mechanisms by which host immunity influences variable HBV outcomes after infection will aid design of novel therapeutic approaches.

As the source of continual HBV production during chronic infection, cccDNA is one of the critical targets for therapeutic modulation [6,13,20]. The optimal treatment outcome is inducing a functional cure, which attempts to recapitulate the fate of HBV in most persons who are infected as adults but then resolve active infection; i.e., cccDNA may still persist in the liver but is silenced and there is seroclearance of serum HBV DNA and HBsAg [6,10,13,21]. As current therapies rarely impact cccDNA, achieving a functional cure will likely require novel immunomodulating therapies given alone or in combination with current antivirals.

## 2. Lambda Interferons

Interferons (IFNs) are a class of cytokines that play a key role in antiviral defense [22,23]. IFNs are amongst the first cytokines produced when host pattern recognition receptors sense pathogen-associated molecular patterns, and IFNs signal in both an autocrine and paracrine fashion [24]. Human IFNs are classified as type-I, type-II or type-III based on varied receptor binding and host cell receptor expression, as reviewed elsewhere [25,26]. Type-III or lambda IFNs (IFNLs) were first discovered in 2003 as a novel family of cytokines that induce a transcriptional program similar to type-I IFNs [23,27,28,29], but by signaling through a distinct receptor complex. 

Type-I IFNs signal through a heterodimeric receptor composed of interferon receptor alpha-1 (IFNAR1) and IFNAR2, which are expressed on the surface of nearly all cells [23,30,31,32]. In contrast, IFNLs signal through a heterodimeric receptor composed of interferon lambda receptor-1 (IFNLR1) and interleukin-10 receptor subunit beta (IL10RB) [28,33,34,35]. IFNLR1 binds IFNLs with specificity and has restricted cellular expression, whereas IL10RB expression is more broadly distributed and also functions as part of the receptor for IL-10, IL-22 and IL-26 [23,33,36,37]. IFNLR1 is expressed primarily on epithelial cells, such as hepatocytes and those found at mucosal surfaces, and on select immune cells, including plasmacytoid dendritic cells and some B-lymphocytes [38,39,40,41]. There are 4 IFNLs (IFNL1, IFNL2, IFNL3, IFNL4) which all signal by binding IFNLR1 resulting in IL10RB recruitment and initiation of a JAK-STAT signaling cascade [25,26]. Signaling results in expression of hundreds of interferon stimulated genes (ISGs), and IFNL signaling has a slower onset and longer duration of action relative to type-I IFN signaling [42,43], in part due to reduced susceptibility of the IFNL signaling complex to negative regulation [43,44]. Notably, a dinucleotide polymorphism within an exon of *IFNL4* imparts differential capacity to make IFNL4 protein, such that some individuals are incapable of making functional IFNL4 [45], whereas all people can make IFNL1, IFNL2, and IFNL3. 

## 3. Role of IFNL Polymorphisms in Hepatitis C Virus Outcomes

The importance of alterations in IFNL signaling were highlighted by clinical studies examining chronic hepatitis C virus (HCV) outcomes. These studies identified a strong association of IFNL polymorphisms with HCV clearance during the acute stage of infection and of achieving HCV cure with type-I IFN-based therapy of chronic infection [46,47,48,49,50,51]. Patients with the genetic capacity to make functional IFNL4 protein, imparted by variability in the rs368234815 dinucleotide nucleotide polymorphism [45], were less likely to clear acute infection and less likely to respond favorably to type-I IFN-based therapy, suggesting ability to make IFNL4 negatively influences HCV outcomes. An alternative explanation for the association of IFNL polymorphisms with HCV outcomes involves variable *IFNL3* mRNA decay due to a functional single nucleotide polymorphism (SNP) (rs4803217) in the 3’ untranslated region of the *IFNL3* transcript [52]. These data provide strong evidence that alterations in IFNL signaling can have functional consequences on outcomes after infection and that modulation of this innate immune pathway could have therapeutic potential for infectious diseases. 

## 4. IFNL Polymorphisms and HBV Clinical Outcomes 

A minority of chronic HBV patients treated with type-I IFNs and/or NRTIs achieve durable suppression of HBsAg production after treatment cessation, thereby achieving seroclearance [14,53,54]. Unfortunately, this favorable clinical outcome occurs infrequently and furthermore, IFN therapy is costly, can cause significant side effects, and must be given for months [14,54]. Understanding the genetic and mechanistic underpinnings of how and why some patients achieve seroclearance could facilitate differentially targeting or modulating IFN therapy to improve outcomes in more people. The strong association of IFNL polymorphisms with HCV outcomes prompted similar evaluations in HBV patient cohorts. 

Several studies in diverse ethnic and geographic cohorts comprised of healthy volunteers, patients who spontaneously cleared HBV infection, and patients who developed chronic HBV infection identified an association of IFNL polymorphisms with HBV infection outcomes [55,56,57]. However, multiple additional studies [58,59,60,61] and meta-analyses did not identify an association [62,63,64,65,66]. Most analyses examined SNPs within IFNL genes, but a case-control study in a Han Chinese cohort composed of 3128 patients (healthy controls, natural HBV clearance, or chronic HBV infection) found an association of *IFNLR1* polymorphisms (rs7525481, rs4649203) with HBV susceptibility [60], which merits further evaluation in independent cohorts. Taken together, there is not a clear and reproducible association of IFNL polymorphisms with HBV infection outcomes, particularly relative to the strong association that was observed with HCV. 

The association of IFNL polymorphisms with differential outcome after IFN-based therapy for chronic HBV infection has also been evaluated. Several studies identified a significant association of IFNL polymorphisms with either HBe antigen (HBeAg) or HBsAg clearance after receipt of IFN-based therapy [67,68,69,70,71,72,73], including several meta-analyses [74,75]. However, multiple additional studies did not identify a significant association [76,77,78,79,80], including several independently conducted meta-analyses (reviewed in [46], [81,82]). Varying genetic and ethnic backgrounds of the study populations, differences in HBV genotype, and sample size may have influenced these disparate results [70,82,83,84]. Taken together, although IFNL polymorphisms correlated with HBV outcomes after IFN-based therapy in some cohorts, this finding was not as robust or consistently observed across studies relative to HCV [46,84]. To date, a definitive genetic or immune mechanism to explain the variable response of HBV patients to type-I IFN treatment remains elusive [12,14], which hampers efforts to rationally design novel therapeutic approaches that are immunomodulatory.

## 5. IFNL Expression in Response to HBV Infection In Vitro and In Vivo 

**Figure 1:**
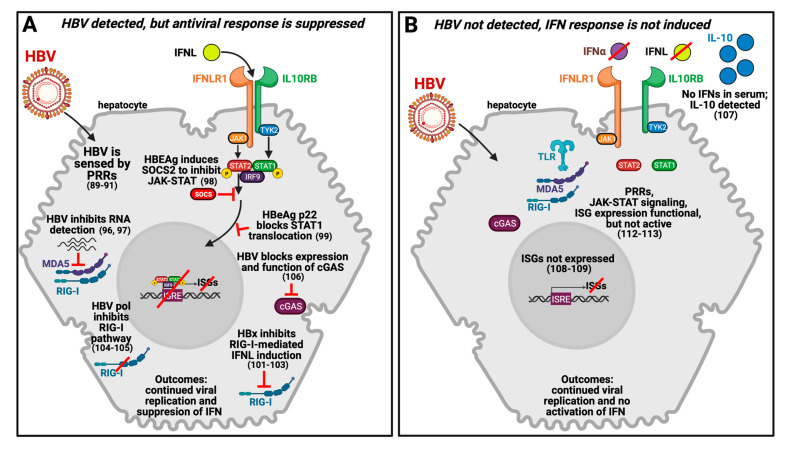
Proposed models and selected mechanisms for HBV suppression or evasion of the host innate immune response in hepatocytes. (**A**) HBV is recognized by the host cell, an antiviral response is induced including expression of IFNLs, but this response is subsequently suppressed by viral elements. (**B**) HBV is not sensed by the host cell and IFNLs are not produced even though detection and signaling proteins are functional. Exposure of HBV-infected hepatocytes to endogenous IFNLs can overcome immune inactivity. Figure created with BioRender.com.

Hepatocytes express IFNLR1 and support IFNL signaling, prompting evaluation of IFNL expression during acute and chronic HBV infection. If HBV infection induces, inhibits, or is responsive to IFNLs, then modulation of IFNL expression endogenously or by exogenous administration could have therapeutic benefit. HBV reactivation during HCV treatment has been observed, likely due to a reduction in hepatic interferon signaling when HCV viral load declines due to antiviral treatment, implying endogenous interferons can suppress HBV infection [85]. The extent to which HBV is recognized within hepatocytes and either stimulates, suppresses, or avoids an innate immune response has differed across studies. These different findings may in part relate to differences in the systems used for evaluation (i.e. in vitro, in vivo, natural infection, or over-expression of viral proteins or virions) [6,86,87,88]. The following section and Figure 1 highlight key investigations providing support for alternative interpretations of how HBV and hepatocytes interact and the potential involvement of IFNLs.

There are two possibilities for how HBV persists within an infected hepatocyte: (1) an antiviral response is induced, but subsequently suppressed, or (2) HBV is a stealth virus and therefore does not induce an IFN response. Viral infection typically is sensed by pathogen-recognition receptors (PRRs) that then induce immune activation. For example, the PRR MDA5 (or IFIH1, interferon induced with helicase C domain 1) is shown to associate with HBV double stranded RNA (dsRNA) [89] and RIG-I (or DDX58, DexD/H-box helicase 58) is reported to recognize a specific region in HBV pre-genomic RNA [90,91]. Upon viral sensing it has been postulated that the resultant innate immune response is subsequently suppressed by HBV or viral components which leads to persistent infection. This result has been shown in vitro in hepatocyte-derived cells lines, primary human hepatocytes, and in vivo in humanized models of HBV infection [90,92,93,94,95].

Studies with HepaRG cells and primary human hepatocytes also showed that HBV can inhibit host cell recognition of viral dsRNA, thus impeding immune signaling [96,97]. Additionally, expression of viral proteins, including the HBV e-antigen (HBeAg) can contribute to immune suppression. HBV infection of human hepatoma cells showed that HBeAg induces a member of the suppressor of cytokine signaling family, SOCS2, which impairs JAK-STAT signaling and leads to downregulation of type-I and IFNL receptors and thus inhibits ISG expression [98]. Moreover, the intracellular form of HBeAg, p22, interferes with IFN signaling in hepatoma cells by blocking nuclear translocation of STAT1 through interactions with the nuclear transport factor, karyopherin alpha1 [99]. These data and others suggest a mechanism by which HBeAg facilitates HBV replication and persistence [100]. 

The HBV protein x (HBx) has also been shown to interfere with multiple host cell functions. Independent studies show that HBx interacts with beta interferon promoter stimulator 1 (IPS-1) adaptor protein and thus interferes with the RIG-I pathway [101], as well as disrupts the assembly and promotes degradation of mitochondrial anti-viral signaling (MAVS) thereby inhibiting IRF3 (interferon regulatory factor 3) activation [102,103]. HBV polymerase has been demonstrated to inhibit the RIG-I pathway by disruption of DDX3 DEAD box RNA helicase [104] or blockade of IRF3 activation [105]. Studies in cell lines and mouse models suggest HBV represses expression and function of the cyclic guanosine monophosphate-adenosine monophosphate synthase (cGAS) DNA sensing pathway [106]. Use of a 3D microfluidic culture model designed to mimic the hepatic sinusoid structure and support long-term HBV infection in vitro showed that HBV suppresses type-I IFNs and IFNL expression in primary human hepatocytes, although the cells remained responsive to exogenous IFN treatment [86]. Collectively, these data imply that HBV is recognized within hepatocytes, an innate immune response is induced and includes expression of IFNLs, however there is subsequent suppression of IFN signaling by viral elements. These data also indicate that modulation of IFNL signaling can impact host cell detection of HBV and affect viral replication, and thus has therapeutic potential. 

Conversely, other studies suggested HBV can act as a stealth virus which avoids IFNL induction during acute infection. In an analysis of peripheral blood from 21 patients with acute HBV infection, no soluble IFN-alpha or IFNL1 was detected during peak viremia, while expression of immunosuppressive IL-10 increased in parallel with an attenuated NK- and T-cell immune response [107]. This result was compatible with findings from a chimpanzee model of HBV infection where no ISG expression was observed in the liver during the early phases of infection, suggesting an IFN response was not triggered in hepatocytes with actively replicating HBV [16,108,109]. HBV may evade DNA sensing by the cGAS and STING (stimulator of interferon genes) pathway within hepatocytes in vivo [106,110]. Other in vitro and in vivo studies also suggest that HBV may not interfere with innate immunity in hepatocytes [85,111,112].

Co-infection studies with HCV or hepatitis D virus (HDV) provided added support that HBV is a stealth virus. HBV-infection of NTCP-expressing human hepatoma cells or primary human hepatocytes did not induce an inflammatory response; conversely mono-infection with HCV or HDV or co-infection with either virus plus HBV resulted in IFN-beta and IFNL expression and induction of ISGs [113,114]. Challenge of humanized mice with HBV and HDV resulted in ISG production and significant IFN-beta and IFNL expression, an outcome not detected in HBV-only infected animals [115]. These studies collectively showed that the target cells and murine models had the capacity to respond to viral challenge and that the lack of IFN induction by HBV was not due to blockade of intracellular pattern recognition receptors (RIG-I, MDA5 or TLR3) or from inhibition of JAK-STAT signaling, but rather was attributed to non-detection (i.e. stealth) based on retained susceptibility to exogenous IFN stimulation [112,113].

The immune response to HBV infection is not restricted solely to hepatocytes. As such, a study examined whether macrophages derived from the THP-1 cell line or human monocytes were as non-responsive as hepatocytes. HBV-exposed macrophages were found to be activated and secreted inflammatory cytokines shortly after challenge, although a high dose of virus (10^2^ genomes/cell) was required to achieve a response [112]. Taken together, the extent to which HBV induces IFNL expression or modulates IFNL signaling may be highly contingent upon the cell type, assay system, and context in which the virus-host interaction is evaluated. 

## 6. IFNL Expression during Chronic HBV Infection 

Viral-host interactions may differ during acute and chronic HBV infection. Baseline differences in endogenous IFN expression associate with outcomes after type-I IFN treatment of chronic HCV infection, as patients with higher baseline hepatic ISG expression are less responsive to type-I IFN treatment [116]. As such, multiple studies evaluated whether IFNL expression is altered during chronic HBV infection. In clinical cohorts, IFNL levels were found to be elevated in serum or liver of chronic HBV patients in some [92,93,117], but not all studies [118,119]. During chronic HBV infection, BDCA3+ dendritic cells are enriched in the liver and produce high levels of IFNL upon exposure to synthetic RNA polyI:C, whereas peripheral BDCA3+ dendritic cells and plasmacytoid dendritic cells are impaired, potentially due to chronic exposure to HBsAg [120,121]. Examination of HBV-infected hepatocytes from liver biopsies of patients with chronic HBV infection showed no elevation of ISG or IFN expression as compared to neighboring non-infected hepatocytes or to hepatocytes collected from patients without HBV infection (most had fatty liver disease) [111]. However, IFN-treatment of HBV-infected cells showed retained capacity to induce an IFN-response in HBV-harboring hepatocytes, including expression of IFNLs in response to Sendai virus or poly I:C exposure [111]. Taken together, while modulation of IFNL expression may occur during chronic HBV infection, altered IFNL signaling does not appear to be a prominent feature within HBV-infected hepatocytes, but may occur in other uninfected liver-resident cells, such as dendritic cells or macrophages. 

Currently approved NRTIs suppress HBV replication and improve hepatic inflammation, and several studies assessed whether they might also impact IFNL expression. Interestingly, HBV patients treated with nucleotide analogues (adefovir, tenofovir) had higher serum IFNL3 levels relative to patients treated with nucleoside analogues (entecavir and lamivudine) [122]. In vitro assays additionally showed a dose-dependent induction of IFNL3 by nucleotide-stimulated cells, induction of ISGs, and inhibition of HBsAg [122]. This suggests induction of IFNLs could contribute to the antiviral efficacy of HBV polymerase inhibition.

While HCV-infected hepatocytes exhibit a robust intracellular IFN response to infection, HBV-infected hepatocytes may not manifest a similar response in spite of high levels of HBV replication, as discussed above. HCV is a flavivirus that is readily detected by pattern recognition receptors and induces IFN production, but expresses viral proteins that actively interfere with the IFN signaling cascade (reviewed in [123]). In contrast, HBV is a hepadnavirus that appears to act more as a stealth virus that avoids detection by pattern recognition receptors and does not induce IFN production in infected hepatocytes, as discussed above. One theory for this lack of response is that hepatocytes intrinsically lack dsDNA sensing mechanisms, and thus HBV can replicate unchecked until surrounding immune cells, such as macrophages, detect high levels of circulating virions and stimulate an innate immune response [112,124]. Alternatively, because viral replication occurs intracellularly within nucleocapsid particles, HBV DNA may be hidden from pattern recognition receptors [3,16]. Differences in how the host detects and interacts with these distinct hepatotropic viruses likely helps explain the strong association of IFNL SNPs with HCV clearance during acute infection but the lack of strong association with HBV outcomes. Furthermore, it is likely that while IFNLs may not be actively or solely produced by hepatocytes during acute and chronic HBV infection, they may be produced by immune or non-parenchymal liver-resident cells resident that influence host immune function and hepatic inflammation. 

## 7. IFNL Impact on HBV Replication and cccDNA 

Although IFNL polymorphisms do not strongly associate with HBV outcomes and although HBV does not clearly prompt IFNL production in hepatocytes, the ability to achieve seroclearance with type-I IFNs prompted interest in exploring the therapeutic potential of IFNLs for HBV. The receptivity of HBV-infected hepatocytes to IFN treatment, as outlined above, suggested IFNLs have the potential to impart a clinically significant antiviral effect. The restricted expression of IFNLR1 raised the specter that IFNL therapy could impact cccDNA while causing less systemic side effects than exogenous type-I IFN therapy. This hypothesis was tested in in vitro and in vivo hepatocyte and HBV infection models in advance of clinical trial testing. 

Type-I IFNs have been shown to lower HBV replication in vitro in cell lines and primary human hepatocytes and in vivo in humanized mouse models of infection. The mechanisms by which type-I IFNs impact cccDNA have been reviewed in detail elsewhere and include transcriptional repression of pregenomic and subgenomic RNA from cccDNA, cccDNA degradation (via APOBEC3A), RNA degradation, histone deacetylation, recruiting transcriptional repressors, lowering the binding of STAT1/2 to cccDNA, and inhibition of pregenomic RNA encapsidation [2,13,125,126,127,128]. 

Although the effect of IFNLs on cccDNA have been less well-characterized, overexpressing or inducing expression of IFNLs in hepatocyte cell lines and murine infection models results in ISG induction and inhibition of HBV replication [38,117,129,130,131,132,133]. IFNL3 treatment reduced HBV transcripts and intracellular DNA in HepG2 2.2.15 cells that have clonally integrated HBV, a phenotype linked to changes in the host transcriptome and proteome [132,134,135]. Exposure of primary human hepatocytes or HepaRG cells to type-I IFNs or IFNLs reduced open-circle and cccDNA by causing cccDNA deamination and degradation, a phenotype attributed to induction of APOBEC deaminases [136]. Activation of stellate cell lines through toll-like receptor 3 resulted in induction of type-I IFNs and IFNLs, which inhibited HBV replication in HepG2 cells transfected with an HBV plasmid [137]. Finally, administration of pegylated IFNL1 in a xenograft model mouse model of human hepatocellular carcinoma led to reduced HBsAg expression in vivo [130].

Taken together, the prevailing evidence indicates that IFNLs are capable of inducing an IFN-response in HBV-infected cells and can lower HBV viral load by reducing HBV transcripts and modulating cccDNA, thus inducing similar intracellular events to type-I IFNs. This suggests targeting cccDNA through modulation of IFNL signaling has the potential to contribute to achieving seroclearance.

## 8. IFNLs as HBV Therapeutics 

Although type-I IFNs have the capacity to impact HBV replication in vitro by modulating cccDNA, as discussed above and as previously reviewed in detail [2,138], the majority of patients treated with type-I IFNs do not have durable HBV DNA and HBsAg seroclearance after treatment [13]. This prompted interest in using IFNLs to treat HBV infection. When pegylated IFNL1 was tested in clinical trials for chronic HCV infection, antiviral activity was demonstrated and treatment was generally well tolerated, although a minority of healthy volunteers and HCV subjects experienced hepatotoxicity, primarily at the greatest tested dose (7.5 μg/kg) [139,140]. Based on these results and the ability of IFNLs to suppress HBV replication in vitro and in animal models, IFNLs were tested in clinical trials of patients with chronic HBV infection.

In a phase 2 trial, pegylated IFNL1 treatment in HBeAg+ chronic HBV subjects led to greater on-treatment decline in HBsAg and HBV DNA relative to patients who received pegylated IFNα2 treatment and similar serologic/virologic responses at the end of treatment [141]. Pegylated IFNL1 treatment was generally well-tolerated, with the exception of a subset of patients who experienced alanine aminotransferase (ALT) flares either during treatment or post-treatment in association with viral rebound [141]. Intriguingly, on-treatment HBV decline was observed in 12 of 13 patients who experienced early ALT flares, implying a mechanistic link between pegylated IFNL1-induced inflammation and antiviral efficacy [141]. Despite these on-treatment results, pegylated IFNL1 did not meet non-inferiority criteria as fewer subjects achieved HBeAg seroconversion relative to the pegylated IFNα2 group 24 weeks post-treatment [141]. In a separate arm of this trial, 13 subjects received twelve weeks of the NRTI entecavir prior to pegylated IFNL1 treatment. Interestingly, in a distinct subset of responders who exhibited HBV DNA and HBsAg decline, treatment with pegylated IFNL1 was found to increase NK-cell polyfunctionality and anti-HBV CD4+ and CD8+ T-cell function [142]. These data suggest differential host response to therapy could have a genetic or biologic basis [142], and that some patients may respond more favorably to IFNL therapy than others, much as has been observed for type-I IFN-based therapy for chronic HCV and HBV infection.

Although there are no current clinical trials evaluating IFNL therapy for HBV mono-infection, pegylated IFNL1 has been investigated for treatment of HDV in patients with HDV/HBV co-infection. Unlike HBV, HDV RNA is recognized by MDA5 in hepatocytes, and induces an innate immune response in cellular systems, which includes induction of type-I IFNs and IFNLs [114,143]. Pegylated IFNL1 was recently tested in combination with ritonavir and lonafarnib (which prevents prenylation of L-HDAg) (Clinical Trials.gov Identifier: NCT03600714) [143] based on studies demonstrating antiviral potential for HDV in murine models [144,145]. 

## 9. Conclusions and Future Directions

In conclusion, the association of IFNL polymorphisms with HBV infection outcomes and response to IFN-based treatment is either absent or weaker than the association observed with HCV. This difference may relate to the distinct ways each virus interfaces with host immunity. The predominance of evidence indicates that HCV stimulates and then interferes with antiviral mechanisms induced by IFNs, while HBV largely avoids stimulation of IFNs within hepatocytes in the first place. Despite reported antagonisms of IFN signaling by HBV, hepatocytes infected with HBV can induce a robust interferon response upon exposure to type-I IFNs and IFNLs, resulting in reduced HBV replication and suppression of cccDNA activity. Therapeutic use of IFNLs alone or in combination with entecavir led to on-treatment viral suppression in HBeAg+ HBV patients and augmented innate and adaptive immune function in a subset of participants, but clinical end points of seroclearance were not achieved, indicating IFNL therapy alone or in combination with entecavir was as-yet inadequate. 

A number of observations suggest there is continued value in understanding the mechanisms and outcomes of IFNL signaling, particularly when considering their potential future use as combination therapy to achieve seroclearance for chronic HBV infection. The four IFNLs do not bind IFNLR1 with equivalent affinity [46,146,147], and some studies suggest IFNL3 induces a more potent ISG transcriptional response relative to IFNL1 [42], the specific IFNL tested in clinical trials [141]. Whether use of a more potent IFNL ligand could result in improved outcomes merits further study, as has been suggested for type-I ligands [148,149]. Differential outcomes after HBV treatment using novel therapies should be evaluated for association with IFNL polymorphisms, given that some are immunostimulatory [20]. Of note, some immune cells express functional IFNLR1, whereas others express a soluble, truncated form of the receptor that does not support canonical signaling and may respond differently to stimulation [150,151,152,153]. Future studies should assess whether focused targeting of IFNL signaling by modulating IFNLs or IFNLR1 on innate and adaptive immune cells could promote antiviral activity against HBV, outside of their impact on hepatocytes harboring HBV. Finally, the use of IFNL mimetics, targeting IFNL activity specifically to the liver, and differential timing of NRTI combined with IFNL therapy merit further evaluation as potential therapeutic strategies. 

In summary, IFNLs are a complex and highly regulated host innate immune defense system that can impact HBV replication and cccDNA. Additional studies are warranted to better understand how to harness the antiviral potential of IFNL signaling for therapeutic benefit in chronic HBV patients. 

## Data Availability

Not applicable.

## References

[1] Liang T.J. (2009). Hepatitis B: The virus and disease. Hepatology.

[2] Xia Y., Guo H. (2020). Hepatitis B virus cccDNA: Formation, regulation and therapeutic potential. Antivir. Res..

[3] Seeger C., Mason W.S. (2015). Molecular biology of hepatitis B virus infection. Virology.

[4] Nassal M. (2015). HBV cccDNA: Viral persistence reservoir and key obstacle for a cure of chronic hepatitis B. Gut.

[5] Marchetti A.L., Guo H. (2020). New Insights on Molecular Mechanism of Hepatitis B Virus Covalently Closed Circular DNA Formation. Cells.

[6] Rehermann B., Thimme R. (2019). Insights From Antiviral Therapy Into Immune Responses to Hepatitis B and C Virus Infection. Gastroenterology.

[7] Rasche A., Sander A.L., Corman V.M., Drexler J.F. (2019). Evolutionary biology of human hepatitis viruses. J. Hepatol..

[8] Block T.M., Guo H., Guo J.T. (2007). Molecular virology of hepatitis B virus for clinicians. Clin. Liver Dis..

[9] Thomas D.L. (2019). Global Elimination of Chronic Hepatitis. N. Engl. J. Med..

[10] Martinez M.G., Testoni B., Zoulim F. (2019). Biological basis for functional cure of chronic hepatitis B. J. Viral. Hepat..

[11] Revill P.A., Chisari F.V., Block J.M., Dandri M., Gehring A.J., Guo H., Hu J., Kramvis A., Lampertico P., Janssen H.L.A. (2019). A global scientific strategy to cure hepatitis B. Lancet Gastroenterol. Hepatol..

[12] Fanning G.C., Zoulim F., Hou J., Bertoletti A. (2019). Therapeutic strategies for hepatitis B virus infection: Towards a cure. Nat. Reviews. Drug Discov..

[13] Xia Y., Liang T.J. (2019). Development of Direct-acting Antiviral and Host-targeting Agents for Treatment of Hepatitis B Virus Infection. Gastroenterology.

[14] Gehring A.J., Protzer U. (2019). Targeting Innate and Adaptive Immune Responses to Cure Chronic HBV Infection. Gastroenterology.

[15] Alter H., Block T., Brown N., Brownstein A., Brosgart C., Chang K.M., Chen P.J., Chisari F.V., Cohen C., El-Serag H. (2018). A research agenda for curing chronic hepatitis B virus infection. Hepatology.

[16] Wieland S., Thimme R., Purcell R.H., Chisari F.V. (2004). Genomic analysis of the host response to hepatitis B virus infection. Proc. Natl. Acad. Sci. USA.

[17] Choi J., Lim Y.S. (2017). Characteristics, Prevention, and Management of Hepatitis B Virus (HBV) Reactivation in HBV-Infected Patients Who Require Immunosuppressive Therapy. J. Infect. Dis..

[18] Paul S., Saxena A., Terrin N., Viveiros K., Balk E.M., Wong J.B. (2016). Hepatitis B Virus Reactivation and Prophylaxis During Solid Tumor Chemotherapy: A Systematic Review and Meta-analysis. Ann. Intern. Med..

[19] Perrillo R.P., Martin P., Lok A.S. (2015). Preventing hepatitis B reactivation due to immunosuppressive drug treatments. JAMA J. Am. Med. Assoc..

[20] Liang T.J., Ghany M.G. (2013). Current and future therapies for hepatitis C virus infection. N. Engl. J. Med..

[21] Cornberg M., Lok A.S., Terrault N.A., Zoulim F., 2019 EASL-AASLD HBV Treatment Endpoints Conference Faculty (2020). Guidance for design and endpoints of clinical trials in chronic hepatitis B—Report from the 2019 EASL-AASLD HBV Treatment Endpoints Conference (double dagger). J. Hepatol.

[22] Borden E.C., Sen G.C., Uze G., Silverman R.H., Ransohoff R.M., Foster G.R., Stark G.R. (2007). Interferons at age 50: Past, current and future impact on biomedicine. Nat. Rev. Drug Discov..

[23] Lin F.C., Young H.A. (2014). Interferons: Success in anti-viral immunotherapy. Cytokine Growth Factor Rev..

[24] Mogensen T.H. (2009). Pathogen recognition and inflammatory signaling in innate immune defenses. Clin. Microbiol. Rev..

[25] Lazear H.M., Schoggins J.W., Diamond M.S. (2019). Shared and Distinct Functions of Type I and Type III Interferons. Immunity.

[26] Stanifer M.L., Guo C., Doldan P., Boulant S. (2020). Importance of Type I and III Interferons at Respiratory and Intestinal Barrier Surfaces. Front. Immunol..

[27] Dumoutier L., Lejeune D., Hor S., Fickenscher H., Renauld J.C. (2003). Cloning of a new type II cytokine receptor activating signal transducer and activator of transcription (STAT)1, STAT2 and STAT3. Biochem. J..

[28] Sheppard P., Kindsvogel W., Xu W., Henderson K., Schlutsmeyer S., Whitmore T.E., Kuestner R., Garrigues U., Birks C., Roraback J. (2003). IL-28, IL-29 and their class II cytokine receptor IL-28R. Nat. Immunol..

[29] Wack A., Terczynska-Dyla E., Hartmann R. (2015). Guarding the frontiers: The biology of type III interferons. Nat. Immunol..

[30] de Weerd N.A., Samarajiwa S.A., Hertzog P.J. (2007). Type I interferon receptors: Biochemistry and biological functions. J. Biol. Chem..

[31] Schreiber G., Piehler J. (2015). The molecular basis for functional plasticity in type I interferon signaling. Trends Immunol..

[32] McNab F., Mayer-Barber K., Sher A., Wack A., O’Garra A. (2015). Type I interferons in infectious disease. Nat. Rev. Immunol..

[33] Donnelly R.P., Kotenko S.V. (2010). Interferon-lambda: A new addition to an old family. J. Interferon Cytokine Res..

[34] Kotenko S.V., Gallagher G., Baurin V.V., Lewis-Antes A., Shen M., Shah N.K., Langer J.A., Sheikh F., Dickensheets H., Donnelly R.P. (2003). IFN-lambdas mediate antiviral protection through a distinct class II cytokine receptor complex. Nat. Immunol..

[35] Mendoza J.L., Schneider W.M., Hoffmann H.H., Vercauteren K., Jude K.M., Xiong A., Moraga I., Horton T.M., Glenn J.S., de Jong Y.P. (2017). The IFN-lambda-IFN-lambdaR1-IL-10Rbeta Complex Reveals Structural Features Underlying Type III IFN Functional Plasticity. Immunity.

[36] Forero A., Ozarkar S., Li H., Lee C.H., Hemann E.A., Nadjsombati M.S., Hendricks M.R., So L., Green R., Roy C.N. (2019). Differential Activation of the Transcription Factor IRF1 Underlies the Distinct Immune Responses Elicited by Type I and Type III Interferons. Immunity.

[37] de Weerd N.A., Nguyen T. (2012). The interferons and their receptors--distribution and regulation. Immunol. Cell Biol..

[38] Doyle S.E., Schreckhise H., Khuu-Duong K., Henderson K., Rosler R., Storey H., Yao L., Liu H., Barahmand-pour F., Sivakumar P. (2006). Interleukin-29 uses a type 1 interferon-like program to promote antiviral responses in human hepatocytes. Hepatology.

[39] Rivera A. (2019). Interferon Lambda’s New Role as Regulator of Neutrophil Function. J. Interferon Cytokine Res..

[40] Sommereyns C., Paul S., Staeheli P., Michiels T. (2008). IFN-lambda (IFN-lambda) is expressed in a tissue-dependent fashion and primarily acts on epithelial cells in vivo. PLoS Pathog..

[41] Ye L., Schnepf D., Staeheli P. (2019). Interferon-lambda orchestrates innate and adaptive mucosal immune responses. Nat. Rev. Immunol..

[42] Bolen C.R., Ding S., Robek M.D., Kleinstein S.H. (2013). Dynamic expression profiling of Type I and Type III Interferon-stimulated hepatocytes reveals a stable hierarchy of gene expression. Hepatology.

[43] Stanifer M.L., Pervolaraki K., Boulant S. (2019). Differential Regulation of Type I and Type III Interferon Signaling. Int. J. Mol. Sci..

[44] Blumer T., Coto-Llerena M., Duong F.H.T., Heim M.H. (2017). SOCS1 is an inducible negative regulator of interferon lambda (IFN-lambda)-induced gene expression in vivo. J. Biol. Chem..

[45] Prokunina-Olsson L., Muchmore B., Tang W., Pfeiffer R.M., Park H., Dickensheets H., Hergott D., Porter-Gill P., Mumy A., Kohaar I. (2013). A variant upstream of IFNL3 (IL28B) creating a new interferon gene IFNL4 is associated with impaired clearance of hepatitis C virus. Nat. Genet..

[46] O’Brien T.R., Yang H.I., Groover S., Jeng W.J. (2019). Genetic Factors That Affect Spontaneous Clearance of Hepatitis C or B Virus, Response to Treatment, and Disease Progression. Gastroenterology.

[47] Chevaliez S., Hezode C., Soulier A., Costes B., Bouvier-Alias M., Rouanet S., Foucher J., Bronowicki J.P., Tran A., Rosa I. (2011). High-dose pegylated interferon-alpha and ribavirin in nonresponder hepatitis C patients and relationship with IL-28B genotype (SYREN trial). Gastroenterology.

[48] Ge D., Fellay J., Thompson A.J., Simon J.S., Shianna K.V., Urban T.J., Heinzen E.L., Qiu P., Bertelsen A.H., Muir A.J. (2009). Genetic variation in IL28B predicts hepatitis C treatment-induced viral clearance. Nature.

[49] Suppiah V., Moldovan M., Ahlenstiel G., Berg T., Weltman M., Abate M.L., Bassendine M., Spengler U., Dore G.J., Powell E. (2009). IL28B is associated with response to chronic hepatitis C interferon-alpha and ribavirin therapy. Nat. Genet..

[50] Tanaka Y., Nishida N., Sugiyama M., Kurosaki M., Matsuura K., Sakamoto N., Nakagawa M., Korenaga M., Hino K., Hige S. (2009). Genome-wide association of IL28B with response to pegylated interferon-alpha and ribavirin therapy for chronic hepatitis C. Nat. Genet..

[51] Thomas D.L., Thio C.L., Martin M.P., Qi Y., Ge D., O’Huigin C., Kidd J., Kidd K., Khakoo S.I., Alexander G. (2009). Genetic variation in IL28B and spontaneous clearance of hepatitis C virus. Nature.

[52] McFarland A.P., Horner S.M., Jarret A., Joslyn R.C., Bindewald E., Shapiro B.A., Delker D.A., Hagedorn C.H., Carrington M., Gale M. (2014). The favorable IFNL3 genotype escapes mRNA decay mediated by AU-rich elements and hepatitis C virus-induced microRNAs. Nat. Immunol..

[53] Fonseca M.A., Ling J.Z.J., Al-Siyabi O., Co-Tanko V., Chan E., Lim S.G. (2020). The efficacy of hepatitis B treatments in achieving HBsAg seroclearance: A systematic review and meta-analysis. J. Viral. Hepat..

[54] Lok A.S., McMahon B.J., Brown R.S., Wong J.B., Ahmed A.T., Farah W., Almasri J., Alahdab F., Benkhadra K., Mouchli M.A. (2016). Antiviral therapy for chronic hepatitis B viral infection in adults: A systematic review and meta-analysis. Hepatology.

[55] Seto W.K., Wong D.K., Kopaniszen M., Proitsi P., Sham P.C., Hung I.F., Fung J., Lai C.L., Yuen M.F. (2013). HLA-DP and IL28B polymorphisms: Influence of host genome on hepatitis B surface antigen seroclearance in chronic hepatitis B. Clin. Infect. Dis..

[56] Karatayli S.C., Bozdayi M., Karatayli E., Ozturk T., Husseini A.A., Albayrak R., Ozkan M., Kalaylioglu Z., Yalcin K., Cinar K. (2015). Interleukin-28 gene polymorphisms may contribute to HBsAg persistence and the development of HBeAg-negative chronic hepatitis B. Liver Int. Off. J. Int. Assoc. Study Liver.

[57] Lee I.C., Lin C.H., Huang Y.H., Huo T.I., Su C.W., Hou M.C., Huang H.C., Lee K.C., Chan C.C., Lin M.W. (2013). IL28B polymorphism correlates with active hepatitis in patients with HBeAg-negative chronic hepatitis B. PLoS ONE.

[58] Heidari Z., Moudi B., Mahmoudzadeh-Sagheb H., Hashemi M. (2016). The Correlation Between Interferon Lambda 3 Gene Polymorphisms and Susceptibility to Hepatitis B Virus Infection. Hepat. Mon..

[59] Ma N., Zhang X., Yu F., Gao P., Fan Q., Liu L., Liu D. (2014). Role of IFN-ks, IFN-ks related genes and the DEPDC5 gene in Hepatitis B virus-related liver disease. J. Viral. Hepat..

[60] Ma N., Zhang X., Yang L., Zhou J., Liu W., Gao X., Yu F., Zheng W., Ding S., Gao P. (2018). Role of Functional IFNL4, IFNLR1, IFNA, IFNAR2 Polymorphisms in Hepatitis B virus-related liver disease in Han Chinese population. J. Viral. Hepat..

[61] Martin M.P., Qi Y., Goedert J.J., Hussain S.K., Kirk G.D., Hoots W.K., Buchbinder S., Carrington M., Thio C.L. (2010). IL28B polymorphism does not determine outcomes of hepatitis B virus or HIV infection. J. Infect. Dis..

[62] Chen J., Wang W., Li X., Xu J. (2015). A meta-analysis of the association between IL28B polymorphisms and infection susceptibility of hepatitis B virus in Asian population. BMC Gastroenterol..

[63] Zhao J., Zhang X., Fang L., Pan H., Shi J. (2020). Association between IL28B Polymorphisms and Outcomes of Hepatitis B Virus Infection: A meta-analysis. BMC Med. Genet..

[64] Tang S., Yue M., Wang J., Zhang Y., Yu R., Su J., Peng Z., Wang J. (2014). Associations of IFN-gamma rs2430561 T/A, IL28B rs12979860 C/T and ERalpha rs2077647 T/C polymorphisms with outcomes of hepatitis B virus infection: A meta-analysis. J. Biomed. Res..

[65] Liao Y., Li Y., Cai B., Chen J., Wang L. (2013). Lack of association between interleukin 28B polymorphisms and spontaneous viral clearance in hepatitis B virus patients. Clin. Infect. Dis..

[66] Lee D.H., Lee J.H., Kim Y.J., Park N.H., Cho Y., Lee Y.B., Yoo J.J., Lee M., Cho Y.Y., Choi W.M. (2014). Relationship between polymorphisms near the IL28B gene and spontaneous HBsAg seroclearance: A systematic review and meta-analysis. J. Viral. Hepat..

[67] Lampertico P., Vigano M., Cheroni C., Facchetti F., Invernizzi F., Valveri V., Soffredini R., Abrignani S., De Francesco R., Colombo M. (2013). IL28B polymorphisms predict interferon-related hepatitis B surface antigen seroclearance in genotype D hepatitis B e antigen-negative patients with chronic hepatitis B. Hepatology.

[68] Wu H., Zhao G., Qian F., Liu K., Xie J., Zhou H., Xu J., Xu Y., Han Y., Xie Q. (2015). Association of IL28B polymorphisms with peginterferon treatment response in Chinese Han patients with HBeAg-positive chronic hepatitis B. Liver Int. Off. J. Int. Assoc. Study Liver.

[69] Wu X., Xin Z., Zhu X., Pan L., Li Z., Li H., Liu Y. (2012). Evaluation of susceptibility locus for response to interferon-alpha based therapy in chronic hepatitis B patients in Chinese. Antivir. Res..

[70] Boglione L., Cusato J., Allegra S., Esposito I., Patti F., Cariti G., Di Perri G., D’Avolio A. (2014). Role of IL28-B polymorphisms in the treatment of chronic hepatitis B HBeAg-negative patients with peginterferon. Antivir. Res..

[71] Domagalski K., Pawlowska M., Zalesna A., Tyczyno M., Skorupa-Klaput M., Tretyn A., Halota W. (2014). The relationship between IL-28B polymorphisms and the response to peginterferon alfa-2a monotherapy in anti-HBe-positive patients with chronic HBV infection. Eur. J. Clin. Microbiol. Infect. Dis..

[72] Sonneveld M.J., Wong V.W., Woltman A.M., Wong G.L., Cakaloglu Y., Zeuzem S., Buster E.H., Uitterlinden A.G., Hansen B.E., Chan H.L. (2012). Polymorphisms near IL28B and serologic response to peginterferon in HBeAg-positive patients with chronic hepatitis B. Gastroenterology.

[73] Galmozzi E., Facchetti F., Grossi G., Loglio A., Vigano M., Lunghi G., Colombo M., Lampertico P. (2018). IFNL4 rs368234815 and rs117648444 variants predict off-treatment HBsAg seroclearance in IFN-treated HBeAg-negative chronic hepatitis B patients. Liver Int. Off. J. Int. Assoc. Study Liver.

[74] Zhao Z., Qin Z., Zhou L., Xiang L., You J., Cao R., Wang H., Wang B., Li M. (2019). The impact of IFNL3 genotype on interferon treatment outcome in patients chronically infected with hepatitis B virus: A meta-analysis. Microb. Pathog..

[75] Lin Z., Zhang J., Ma X., Yang S., Tian N., Lin X., Zhou S., Liu L., Gao Y. (2016). The Role of Interferon Lambda 3 Genetic Polymorphisms in Response to Interferon Therapy in Chronic Hepatitis B Patients: An Updated Meta-Analysis. Hepat. Mon..

[76] Cheng L., Sun X., Tan S., Tan W., Dan Y., Zhou Y., Mao Q., Deng G. (2014). Effect of HLA-DP and IL28B gene polymorphisms on response to interferon treatment in hepatitis B e-antigen seropositive chronic hepatitis B patients. Hepatol. Res..

[77] de Niet A., Takkenberg R.B., Benayed R., Riley-Gillis B., Weegink C.J., Zaaijer H.L., Koot M., Jansen P.L., Beld M.G., Lopatin U. (2012). Genetic variation in IL28B and treatment outcome in HBeAg-positive and -negative chronic hepatitis B patients treated with Peg interferon alfa-2a and adefovir. Scand. J. Gastroenterol..

[78] Holmes J.A., Nguyen T., Ratnam D., Heerasing N.M., Tehan J.V., Bonanzinga S., Dev A., Bell S., Pianko S., Chen R. (2013). IL28B genotype is not useful for predicting treatment outcome in Asian chronic hepatitis B patients treated with pegylated interferon-alpha. J. Gastroenterol. Hepatol..

[79] Zhang Q., Lapalus M., Asselah T., Laouenan C., Moucari R., Martinot-Peignoux M., Bieche I., Estrabaud E., De Muynck S., Boyer N. (2014). IFNL3 (IL28B) polymorphism does not predict long-term response to interferon therapy in HBeAg-positive chronic hepatitis B patients. J. Viral. Hepat..

[80] Wei L., Wedemeyer H., Liaw Y.F., Chan H.L., Piratvisuth T., Marcellin P., Jia J., Tan D., Chow W.C., Brunetto M.R. (2018). No association between IFNL3 (IL28B) genotype and response to peginterferon alfa-2a in HBeAg-positive or -negative chronic hepatitis B. PLoS ONE.

[81] Galmozzi E., Vigano M., Lampertico P. (2014). Systematic review with meta-analysis: Do interferon lambda 3 polymorphisms predict the outcome of interferon-therapy in hepatitis B infection?. Aliment. Pharm..

[82] Stattermayer A.F., Ferenci P. (2015). Effect of IL28B genotype on hepatitis B and C virus infection. Curr. Opin. Virol..

[83] Sonneveld M.J., Brouwer W.P., Janssen H.L. (2013). Studies of IL28B genotype and response to peginterferon in chronic hepatitis B should be stratified by HBV genotype. Hepatology.

[84] Jilg N., Chung R.T. (2013). One more piece in the interleukin 28B gene puzzle? The case of hepatitis B. Hepatology.

[85] Cheng X., Uchida T., Xia Y., Umarova R., Liu C.J., Chen P.J., Gaggar A., Suri V., Mucke M.M., Vermehren J. (2020). Diminished hepatic IFN response following HCV clearance triggers HBV reactivation in coinfection. J. Clin. Investig..

[86] Ortega-Prieto A.M., Skelton J.K., Wai S.N., Large E., Lussignol M., Vizcay-Barrena G., Hughes D., Fleck R.A., Thursz M., Catanese M.T. (2018). 3D microfluidic liver cultures as a physiological preclinical tool for hepatitis B virus infection. Nat. Commun..

[87] Verrier E.R., Colpitts C.C., Schuster C., Zeisel M.B., Baumert T.F. (2016). Cell Culture Models for the Investigation of Hepatitis B and D Virus Infection. Viruses.

[88] Hu J., Lin Y.Y., Chen P.J., Watashi K., Wakita T. (2019). Cell and Animal Models for Studying Hepatitis B Virus Infection and Drug Development. Gastroenterology.

[89] Lu H.L., Liao F. (2013). Melanoma differentiation-associated gene 5 senses hepatitis B virus and activates innate immune signaling to suppress virus replication. J. Immunol..

[90] Sato S., Li K., Kameyama T., Hayashi T., Ishida Y., Murakami S., Watanabe T., Iijima S., Sakurai Y., Watashi K. (2015). The RNA sensor RIG-I dually functions as an innate sensor and direct antiviral factor for hepatitis B virus. Immunity.

[91] Khan M., Syed G.H., Kim S.J., Siddiqui A. (2016). Hepatitis B Virus-Induced Parkin-Dependent Recruitment of Linear Ubiquitin Assembly Complex (LUBAC) to Mitochondria and Attenuation of Innate Immunity. PLoS Pathog..

[92] Yu Y., Gong R., Mu Y., Chen Y., Zhu C., Sun Z., Chen M., Liu Y., Zhu Y., Wu J. (2011). Hepatitis B virus induces a novel inflammation network involving three inflammatory factors, IL-29, IL-8, and cyclooxygenase-2. J. Immunol..

[93] Li Y., Xie J., Xu X., Liu L., Wan Y., Liu Y., Zhu C., Zhu Y. (2013). Inducible interleukin 32 (IL-32) exerts extensive antiviral function via selective stimulation of interferon lambda1 (IFN-lambda1). J. Biol. Chem..

[94] Foster G.R., Ackrill A.M., Goldin R.D., Kerr I.M., Thomas H.C., Stark G.R. (1991). Expression of the terminal protein region of hepatitis B virus inhibits cellular responses to interferons alpha and gamma and double-stranded RNA. Proc. Natl. Acad. Sci. USA.

[95] Lutgehetmann M., Bornscheuer T., Volz T., Allweiss L., Bockmann J.H., Pollok J.M., Lohse A.W., Petersen J., Dandri M. (2011). Hepatitis B virus limits response of human hepatocytes to interferon-alpha in chimeric mice. Gastroenterology.

[96] Luangsay S., Gruffaz M., Isorce N., Testoni B., Michelet M., Faure-Dupuy S., Maadadi S., Ait-Goughoulte M., Parent R., Rivoire M. (2015). Early inhibition of hepatocyte innate responses by hepatitis B virus. J. Hepatol..

[97] Lucifora J., Durantel D., Testoni B., Hantz O., Levrero M., Zoulim F. (2010). Control of hepatitis B virus replication by innate response of HepaRG cells. Hepatology.

[98] Yu Y., Wan P., Cao Y., Zhang W., Chen J., Tan L., Wang Y., Sun Z., Zhang Q., Wan Y. (2017). Hepatitis B Virus e Antigen Activates the Suppressor of Cytokine Signaling 2 to Repress Interferon Action. Sci. Rep..

[99] Mitra B., Wang J., Kim E.S., Mao R., Dong M., Liu Y., Zhang J., Guo H. (2019). Hepatitis B Virus Precore Protein p22 Inhibits Alpha Interferon Signaling by Blocking STAT Nuclear Translocation. J. Virol..

[100] Lang T., Lo C., Skinner N., Locarnini S., Visvanathan K., Mansell A. (2011). The hepatitis B e antigen (HBeAg) targets and suppresses activation of the toll-like receptor signaling pathway. J. Hepatol..

[101] Kumar M., Jung S.Y., Hodgson A.J., Madden C.R., Qin J., Slagle B.L. (2011). Hepatitis B virus regulatory HBx protein binds to adaptor protein IPS-1 and inhibits the activation of beta interferon. J. Virol..

[102] Wang X., Li Y., Mao A., Li C., Li Y., Tien P. (2010). Hepatitis B virus X protein suppresses virus-triggered IRF3 activation and IFN-beta induction by disrupting the VISA-associated complex. Cell Mol. Immunol..

[103] Wei C., Ni C., Song T., Liu Y., Yang X., Zheng Z., Jia Y., Yuan Y., Guan K., Xu Y. (2010). The hepatitis B virus X protein disrupts innate immunity by downregulating mitochondrial antiviral signaling protein. J. Immunol..

[104] Wang H., Ryu W.S. (2010). Hepatitis B virus polymerase blocks pattern recognition receptor signaling via interaction with DDX3: Implications for immune evasion. PLoS Pathog..

[105] Yu S., Chen J., Wu M., Chen H., Kato N., Yuan Z. (2010). Hepatitis B virus polymerase inhibits RIG-I- and Toll-like receptor 3-mediated beta interferon induction in human hepatocytes through interference with interferon regulatory factor 3 activation and dampening of the interaction between TBK1/IKKepsilon and DDX3. J. Gen. Virol..

[106] Verrier E.R., Yim S.A., Heydmann L., El Saghire H., Bach C., Turon-Lagot V., Mailly L., Durand S.C., Lucifora J., Durantel D. (2018). Hepatitis B Virus Evasion from Cyclic Guanosine Monophosphate-Adenosine Monophosphate Synthase Sensing in Human Hepatocytes. Hepatology.

[107] Dunn C., Peppa D., Khanna P., Nebbia G., Jones M., Brendish N., Lascar R.M., Brown D., Gilson R.J., Tedder R.J. (2009). Temporal analysis of early immune responses in patients with acute hepatitis B virus infection. Gastroenterology.

[108] Yoshio S., Kanto T. (2016). Host-virus interactions in hepatitis B and hepatitis C infection. J. Gastroenterol..

[109] Wieland S.F., Chisari F.V. (2005). Stealth and cunning: Hepatitis B and hepatitis C viruses. J. Virol..

[110] Lauterbach-Riviere L., Bergez M., Monch S., Qu B., Riess M., Vondran F.W.R., Liese J., Hornung V., Urban S., Konig R. (2020). Hepatitis B Virus DNA is a Substrate for the cGAS/STING Pathway but is not Sensed in Infected Hepatocytes. Viruses.

[111] Suslov A., Boldanova T., Wang X., Wieland S., Heim M.H. (2018). Hepatitis B Virus Does Not Interfere with Innate Immune Responses in the Human Liver. Gastroenterology.

[112] Cheng X., Xia Y., Serti E., Block P.D., Chung M., Chayama K., Rehermann B., Liang T.J. (2017). Hepatitis B virus evades innate immunity of hepatocytes but activates cytokine production by macrophages. Hepatology.

[113] Mutz P., Metz P., Lempp F.A., Bender S., Qu B., Schoneweis K., Seitz S., Tu T., Restuccia A., Frankish J. (2018). HBV Bypasses the Innate Immune Response and Does Not Protect HCV From Antiviral Activity of Interferon. Gastroenterology.

[114] Zhang Z., Filzmayer C., Ni Y., Sultmann H., Mutz P., Hiet M.S., Vondran F.W.R., Bartenschlager R., Urban S. (2018). Hepatitis D virus replication is sensed by MDA5 and induces IFN-beta/lambda responses in hepatocytes. J. Hepatol..

[115] Giersch K., Allweiss L., Volz T., Helbig M., Bierwolf J., Lohse A.W., Pollok J.M., Petersen J., Dandri M., Lutgehetmann M. (2015). Hepatitis Delta co-infection in humanized mice leads to pronounced induction of innate immune responses in comparison to HBV mono-infection. J. Hepatol..

[116] Dill M.T., Duong F.H., Vogt J.E., Bibert S., Bochud P.Y., Terracciano L., Papassotiropoulos A., Roth V., Heim M.H. (2011). Interferon-induced gene expression is a stronger predictor of treatment response than IL28B genotype in patients with hepatitis C. Gastroenterology.

[117] Cao Y., Zhang R., Zhang W., Zhu C., Yu Y., Song Y., Wang Q., Bai L., Liu Y., Wu K. (2014). IL-27, a cytokine, and IFN-lambda1, a type III IFN, are coordinated to regulate virus replication through type I IFN. J. Immunol..

[118] Shi X., Chi X., Pan Y., Gao Y., Li W., Yang C., Zhong J., Xu D., Zhang M., Minuk G. (2015). IL28B is associated with outcomes of chronic HBV infection. Yonsei Med. J..

[119] de Groen R.A., McPhee F., Friborg J., Janssen H.L., Boonstra A. (2014). Endogenous IFNlambda in viral hepatitis patients. J. Interferon Cytokine Res..

[120] van der Aa E., Buschow S.I., Biesta P.J., Janssen H.L., Woltman A.M. (2016). The Effect of Chronic Hepatitis B Virus Infection on BDCA3+ Dendritic Cell Frequency and Function. PLoS ONE.

[121] van der Molen R.G., Sprengers D., Binda R.S., de Jong E.C., Niesters H.G., Kusters J.G., Kwekkeboom J., Janssen H.L. (2004). Functional impairment of myeloid and plasmacytoid dendritic cells of patients with chronic hepatitis B. Hepatology.

[122] Murata K., Asano M., Matsumoto A., Sugiyama M., Nishida N., Tanaka E., Inoue T., Sakamoto M., Enomoto N., Shirasaki T. (2018). Induction of IFN-lambda3 as an additional effect of nucleotide, not nucleoside, analogues: A new potential target for HBV infection. Gut.

[123] Pagliaccetti N.E., Chu E.N., Bolen C.R., Kleinstein S.H., Robek M.D. (2010). Lambda and alpha interferons inhibit hepatitis B virus replication through a common molecular mechanism but with different in vivo activities. Virology.

[124] Tan G., Song H., Xu F., Cheng G. (2018). When Hepatitis B Virus Meets Interferons. Front. Microbiol..

[125] Belloni L., Allweiss L., Guerrieri F., Pediconi N., Volz T., Pollicino T., Petersen J., Raimondo G., Dandri M., Levrero M. (2012). IFN-alpha inhibits HBV transcription and replication in cell culture and in humanized mice by targeting the epigenetic regulation of the nuclear cccDNA minichromosome. J. Clin. Investig..

[126] Wang Y.X., Niklasch M., Liu T., Wang Y., Shi B., Yuan W., Baumert T.F., Yuan Z., Tong S., Nassal M. (2020). Interferon-inducible MX2 is a host restriction factor of hepatitis B virus replication. J. Hepatol..

[127] Yang Y., Zhao X., Wang Z., Shu W., Li L., Li Y., Guo Z., Gao B., Xiong S. (2020). Nuclear Sensor Interferon-Inducible Protein 16 Inhibits the Function of Hepatitis B Virus Covalently Closed Circular DNA by Integrating Innate Immune Activation and Epigenetic Suppression. Hepatology.

[128] Robek M.D., Boyd B.S., Wieland S.F., Chisari F.V. (2004). Signal transduction pathways that inhibit hepatitis B virus replication. Proc. Natl. Acad. Sci. USA.

[129] Kanda T., Jiang X., Nakamoto S., Nakamura M., Miyamura T., Wu S., Yokosuka O. (2013). Different effects of three interferons L on Toll-like receptor-related gene expression in HepG2 cells. Cytokine.

[130] Tian S., Hui X., Fan Z., Li Q., Zhang J., Yang X., Ma X., Huang B., Chen D., Chen H. (2014). Suppression of hepatocellular carcinoma proliferation and hepatitis B surface antigen secretion with interferon-lambda1 or PEG-interferon-lambda1. FASEB J..

[131] Robek M.D., Boyd B.S., Chisari F.V. (2005). Lambda interferon inhibits hepatitis B and C virus replication. J. Virol..

[132] Guo X., Chen D., Cai Q., Huang Z., Xu W., Peng L., Chen P. (2020). Minicircle DNA vector expressing interferon-lambda-3 inhibits hepatitis B virus replication and expression in hepatocyte-derived cell line. BMC Mol. Cell Biol..

[133] Nakagawa S., Hirata Y., Kameyama T., Tokunaga Y., Nishito Y., Hirabayashi K., Yano J., Ochiya T., Tateno C., Tanaka Y. (2013). Targeted induction of interferon-lambda in humanized chimeric mouse liver abrogates hepatotropic virus infection. PLoS ONE.

[134] Makjaroen J., Somparn P., Hodge K., Poomipak W., Hirankarn N., Pisitkun T. (2018). Comprehensive Proteomics Identification of IFN-lambda3-regulated Antiviral Proteins in HBV-transfected Cells. Mol. Cell Proteom..

[135] Hodge K., Makjaroen J., Robinson J., Khoomrung S., Pisitkun T. (2020). Deep Proteomic Deconvolution of Interferons and HBV Transfection Effects on a Hepatoblastoma Cell Line. ACS Omega.

[136] Bockmann J.H., Stadler D., Xia Y., Ko C., Wettengel J.M., Schulze Zur Wiesch J., Dandri M., Protzer U. (2019). Comparative Analysis of the Antiviral Effects Mediated by Type I and III Interferons in Hepatitis B Virus-Infected Hepatocytes. J. Infect. Dis..

[137] Zhang B., Liu Y., Wang X., Li J., Xu X., Guo L., Ho W.Z. (2018). TLR3 Activation of Hepatic Stellate Cell Line Suppresses HBV Replication in HepG2 Cells. Front. Immunol..

[138] Mitra B., Thapa R.J., Guo H., Block T.M. (2018). Host functions used by hepatitis B virus to complete its life cycle: Implications for developing host-targeting agents to treat chronic hepatitis B. Antivir. Res..

[139] Muir A.J., Shiffman M.L., Zaman A., Yoffe B., de la Torre A., Flamm S., Gordon S.C., Marotta P., Vierling J.M., Lopez-Talavera J.C. (2010). Phase 1b study of pegylated interferon lambda 1 with or without ribavirin in patients with chronic genotype 1 hepatitis C virus infection. Hepatology.

[140] Ramos E.L. (2010). Preclinical and clinical development of pegylated interferon-lambda 1 in chronic hepatitis C. J. Interferon Cytokine Res..

[141] Chan H.L.Y., Ahn S.H., Chang T.T., Peng C.Y., Wong D., Coffin C.S., Lim S.G., Chen P.J., Janssen H.L.A., Marcellin P. (2016). Peginterferon lambda for the treatment of HBeAg-positive chronic hepatitis B: A randomized phase 2b study (LIRA-B). J. Hepatol..

[142] Phillips S., Mistry S., Riva A., Cooksley H., Hadzhiolova-Lebeau T., Plavova S., Katzarov K., Simonova M., Zeuzem S., Woffendin C. (2017). Peg-Interferon Lambda Treatment Induces Robust Innate and Adaptive Immunity in Chronic Hepatitis B Patients. Front. Immunol..

[143] Zhang Z., Urban S. (2020). New insights into HDV persistence: The role of interferon response and implications for upcoming novel therapies. J. Hepatol..

[144] Koh C., Da B.L., Glenn J.S. (2019). HBV/HDV Coinfection: A Challenge for Therapeutics. Clin. Liver Dis..

[145] Giersch K., Homs M., Volz T., Helbig M., Allweiss L., Lohse A.W., Petersen J., Buti M., Pollicino T., Sureau C. (2017). Both interferon alpha and lambda can reduce all intrahepatic HDV infection markers in HBV/HDV infected humanized mice. Sci. Rep..

[146] Egli A., Santer D.M., O’Shea D., Tyrrell D.L., Houghton M. (2014). The impact of the interferon-lambda family on the innate and adaptive immune response to viral infections. Emerg. Microbes Infect..

[147] Syedbasha M., Linnik J., Santer D., O’Shea D., Barakat K., Joyce M., Khanna N., Tyrrell D.L., Houghton M., Egli A. (2016). An ELISA Based Binding and Competition Method to Rapidly Determine Ligand-receptor Interactions. J. Vis. Exp..

[148] Hillyer P., Mane V.P., Schramm L.M., Puig M., Verthelyi D., Chen A., Zhao Z., Navarro M.B., Kirschman K.D., Bykadi S. (2012). Expression profiles of human interferon-alpha and interferon-lambda subtypes are ligand- and cell-dependent. Immunol. Cell Biol..

[149] Chen J., Li Y., Lai F., Wang Y., Sutter K., Dittmer U., Ye J., Zai W., Liu M., Shen F. (2020). Functional Comparison of IFN-alpha Subtypes Reveals Potent HBV Suppression by a Concerted Action of IFN-alpha and -gamma Signaling. Hepatology.

[150] Liu B.S., Janssen H.L., Boonstra A. (2012). Type I and III interferons enhance IL-10R expression on human monocytes and macrophages, resulting in IL-10-mediated suppression of TLR-induced IL-12. Eur. J. Immunol..

[151] Santer D.M., Minty G.E.S., Golec D.P., Lu J., May J., Namdar A., Shah J., Elahi S., Proud D., Joyce M. (2020). Differential expression of interferon-lambda receptor 1 splice variants determines the magnitude of the antiviral response induced by interferon-lambda 3 in human immune cells. PLoS Pathog..

[152] De M., Bhushan A., Chinnaswamy S. (2020). Monocytes differentiated into macrophages and dendritic cells in the presence of human IFN-lambda3 or IFN-lambda4 show distinct phenotypes. J. Leukoc. Biol..

[153] Witte K., Gruetz G., Volk H.D., Looman A.C., Asadullah K., Sterry W., Sabat R., Wolk K. (2009). Despite IFN-lambda receptor expression, blood immune cells, but not keratinocytes or melanocytes, have an impaired response to type III interferons: Implications for therapeutic applications of these cytokines. Genes Immun..

